# Willingness to Pay for Complementary Health Care Insurance in Iran

**Published:** 2017-09

**Authors:** Shirin NOSRATNEJAD, Arash RASHIDIAN, Ali AKBARI SARI, Najme MORADI

**Affiliations:** 1. Tabriz Health Services Management Research Center, Dept. of Health Services Management, School of Management and Medical Informatics, Tabriz University of Medical Sciences, Tabriz, Iran; 2. Iranian Center of Excellence in Health Services Management, School of Management and Medical Informatics, Tabriz University of Medical Sciences, Tabriz, Iran; 3. Dept. of Health Management and Economics, School of Public Health, Tehran University of Medical Sciences, Tehran, Iran; 4. Food and Drug Administration, Ministry of Health and Medical Education, Tehran, Iran

**Keywords:** Willingness to pay, Health insurance, Contingent valuation method, Iran

## Abstract

**Background:**

Complementary health insurance is increasingly used to remedy the limitations and shortcomings of the basic health insurance benefit packages. Hence, it is essential to gather reliable information about the amount of Willingness to Pay (WTP) for health insurance. We assessed the WTP for health insurance in Iran in order to suggest an affordable complementary health insurance.

**Methods:**

The study sample consisted of 300 household heads all over provinces of Iran in 2013. The method applied was double bounded dichotomous choice and open-ended question approach of contingent valuation.

**Results:**

The average WTP for complementary health insurance per person per month by double bounded dichotomous choice and open-ended question method respectively was 199000 and 115300 Rials (8 and 4.6 USD, respectively). Household’s heads with higher levels of income and those who worked had more WTP for the health insurance. Besides, the WTP increased in direct proportion to the number of insured members of each household and in inverse proportion to the family size.

**Conclusion:**

The WTP value can be used as a premium in a society. As an important finding, the study indicated that the households were willing to pay higher premiums than currently collected for the complementary health insurance coverage in Iran. This offers the policy makers the opportunity to increase the premium and provide good benefits package for insured people of country then better risk pooling.

## Introduction

Policy makers consider three broad options for financing health care in contrast to catastrophic effects of illnesses; taxation, social security and private health insurance (1) unlike taxation and social security viewed as tools for improving equity, private insurance viewed as unequal access, large number of uninsured people and suitability only for rich young persons. Evidence confirmed unregulated or poorly design of private health insurance could result in inequalities such as covering healthy and rich persons and escalating costs (1).

Private health insurance may deliver primary and secondary health coverage. Primary coverage often covers the broad range of services afforded through public financing. Secondary coverage completes the ones provided by social or publicly funded package or even covers some costs such as co-payments and services like dental care or outpatient drugs not provided by basic health insurance. Then, the services of private health insurance are divided into four categories: primary, duplicate, complementary, and supplementary health care services. The roles of private health insurance are different among countries and depended on the each country’s wealth and institutional development. In many countries, the health care reforms of two decades ago have used private health insurance as a way for gathering private funds for health care (1).

If the private health care insurance were appropriately managed, it could play an important role on health care access, especially in developing countries, because, in those countries, the out of pocket payments are the most common form of financing which causes huge financing burden on households. In addition, in poorer countries, collection of tax revenue is very difficult, since, many people work in private sectors. Thus, the ability of government for support of broad health care services is limited. Then, private health insurance continues to be important even in countries with universal coverage, because, it supplements or completes the basic benefit package offered by mandatory health insurance and decreases the out of pocket payment (1). In addition, most countries have some kind of private health insurance (1), but there are limited data on the private health insurance expenditure, the population under coverage of this insurance, and its premium (1).

This study used the data of households for estimation the willingness to pay for private health insurance in Iran, applying a Contingent Valuation Method (CVM). Moreover, we tried to determinate the variables that affected the people’s willingness to pay. This helps policymakers for expanding private health insurance

## Methods

We applied the double-bounded dichotomous choices (also known as ‘referendum format’) and open-ended question method to estimate the WTP for complementary health insurance in Iran. In dichotomous choice methods, the respondent only answers ‘yes’ or ‘no’ to a given two questions about the WTP amount. The first question would be followed by another question specifying a lower amount, if the answer to the first question were negative, or a higher amount, to positive answer (2). To avoid the initial bid bias, we used four different starting bids

In this method, the respondents’ answers were divided into four groups: “yes, yes”, “yes, no”, “no, yes” and “no, no”. Comparing this method with other elicitation methods, this procedure was evaluated to have the most significant statistical efficiency (3, 4).

In open-ended questions, the simplest method of CVM, the respondent is open to say any amount that he/she wants.

### Sample size and data collection:

Given that, the probability of having complementary health care insurance by an Iranian household was about 15% (5), and to assure interval of 95% and a power of test of 80%. The sample size of the study should include 200 households, but since the study was cross-sectional, we considered a sample size of 300 households to avoid the sample attrition resulting from the questionnaires that did not respond. We included the households that already covered by any mandatory or voluntary basic health insurance coverage. We interviewed a household head (male or female) willing to participate in the study.

[1] $$\begin{array}{l} p=0.15\\ (1-p)=0.85\\ d=0.05\\ n=\frac{Z_{1-\frac{\alpha }{2}}^{2}p(1-p)}{d^{2}}=\frac{{\left(1.96\right)}^{2}(0.15)(0.85)}{{\left(0.05\right)}^{2}}=200 \end{array}$$

This was a cross-sectional study and a questionnaire survey was administrated to the main study sample including 300 households in all Iranian provinces randomly chosen from among the 27000 households with basic health insurance coverage, as indicated by the 2010 Iran multiple-indicator Demographic and Health Survey (5). The data collection method was via a telephone interview with household heads. The heads of the selected households were interviewed in Sep 2013 by two trained interviewers.

The respondents’ names or other characteristics were not identified after interviews were recorded. We received ethical approval for conducting this study.

### Questionnaire

The questionnaire, designed according to the guideline of the Contingent Valuation (CV) studies (2), was a structured questionnaire. Validity and reliability of the CV questionnaire have been confirmed repeatedly (4, 6). The questionnaire consisted of four parts. In the first part, the interviewers described the purpose of the study and asked each respondent whether they had health care insurance coverage. In the following part, the respondents answered the hypothetical scenarios of CV by two methods of double bonded dichotomous choice and open-ended questions. We calculated the bid using a pilot study and the actual premiums the private health insurance companies were requesting at the time of the study and using the result of last study in Iran (7). We then randomly distributed different starting values among the respondents to avoid starting point bias (8). In the third part, each household head was asked about the health status of themselves and their family members. The final part included the socio-demographic questions. Neither respondents’ names nor their particulars were identified after the interviews recorded.

We received ethical approval for conducting this study. We obtained an informed consent from each participant taking part in this study at the start of the telephone interview.

### Econometric model

In double-bounded dichotomous choice method, we asked two questions with two answers of yes or no, then we had four kinds of responses to the WTP questions: (A) ‘yes’ – ‘no’, (B) ‘yes’ – ‘yes’, (C) ‘no’ – ‘yes’, (D) ‘no’ – ‘no’.

The function to be estimated is:

[2] $${\mathrm{WTP}}_{i}\left(z_{i},u_{i}\right)=z_{i}^{\prime }\beta +u_{i}\;\;\;\mathrm{and}\;\;\;u_{i}\approx N(0,\sigma^{2})$$

Afterward, the probability of each of the four cases is defined as:

*
(A)* :
  [3] $$\text{Pr}\left(t^{1}\leq \mathrm{WTP}<t^{2}\right)=\text{Pr}\left(\frac{t^{1}-z_{i}^{\prime }\beta }{\sigma }\leq \frac{u_{i}}{\sigma }<\frac{t^{2}-z_{i}^{\prime }\beta }{\sigma }\right)=\Phi \left(z_{i}^{\prime }\frac{\beta }{\sigma }-\frac{t^{1}}{\sigma }\right)-\Phi \left(z_{i}^{\prime }\frac{\beta }{\sigma }-\frac{t^{2}}{\sigma }\right)$$
*
(B) *:
  [4] $$\text{Pr}(\mathrm{WTP}>t^{1},\mathrm{WTP}>t^{2})=\Phi \left(z_{i}^{\prime }\frac{\beta }{\sigma }-\frac{t^{2}}{\sigma }\right)$$
*
(C)* :
  [5] $$\text{Pr}\left(t^{2}\leq \mathrm{WTP}<t^{1}\right)=\Phi \left(z_{i}^{\prime }\frac{\beta }{\sigma }-\frac{t^{2}}{\sigma }\right)-\Phi \left(z_{i}^{\prime }\frac{\beta }{\sigma }-\frac{t^{1}}{\sigma }\right)$$
*
(D)* :
  [6] $$\text{Pr}(\mathrm{WTP}<t^{1},\mathrm{WTP}<t^{2})=1-\Phi \left(z_{i}^{\prime }\frac{\beta }{\sigma }-\frac{t^{2}}{\sigma }\right)$$

Estimation of *β* and *σ* were based on maximum likelihood method. The function that needs to be maximized to find the parameters of the model is:

$$\sum_{i=1}^{N}\left[\begin{array}{c} d_{i}^{\mathrm{YN}}\ln\left(\Phi \left(z_{i}^{\prime }\frac{\beta }{\sigma }-\frac{t^{1}}{\sigma }\right)-\Phi \left(z_{i}^{\prime }\frac{\beta }{\sigma }-\frac{t^{2}}{\sigma }\right)\right)+d_{i}^{\mathrm{YY}}\ln\left(\Phi \left(z_{i}^{\prime }\frac{\beta }{\sigma }-\frac{t^{2}}{\sigma }\right)\right)+\\ d_{i}^{\mathrm{NY}}\ln\left(\Phi \left(z_{i}^{\prime }\frac{\beta }{\sigma }-\frac{t^{2}}{\sigma }\right)-\Phi \left(z_{i}^{\prime }\frac{\beta }{\sigma }-\frac{t^{1}}{\sigma }\right)\right)+d_{i}^{\mathrm{NN}}\ln\left(1-\Phi \left(z_{i}^{\prime }\frac{\beta }{\sigma }-\frac{t^{2}}{\sigma }\right)\right) \end{array}\right]$$

Moreover, $d_{i}^{\mathrm{YN}}$ , $d_{i}^{\mathrm{YY}}$ , $d_{i}^{\mathrm{NY}}$ , $d_{i}^{\mathrm{NN}}$ are indicator variables that take the value of one or zero, depending on the relevant case for each individual. Household heads contribute to the logarithm of the likelihood function in only one of its four parts. Here, we obtained directly *β* and *σ*. Then, we can estimate WTP (9).

For estimating WTP by open-ended question method, we used the 1% trimmed mean.

## Results

The summary statistics of explanatory variable are presented in Table 1.

**Table 1:** The summery statistics of explanatory variables

**Variable name**	**Description**	**Mean**	**Std. Dev.**	**Number (Percent)**
**Gender**	Indicated the gender of each household’s head: 1 for a male, 0 for a female			(91)
**Family size**	Indicated the number of people in each family	3.67	1.48	
**experience**	Indicated previous use of health insurance:			120 (40)
	1 if insurance had been used in the past,			
	0 if not			
**Insured members**	Indicated the number of insured members in each family	1.19	1.7	
**Excellent health status**	Indicated the health status of each household’s head (a self-report variable): 1 if health status were excellent, 0 if otherwise			(26)
**Good health**	Indicated the health status of each household head (a self-report variable), 1 if health status were good, 0 if otherwise			150 (50)
**Middle health**	Indicated the health status of each household head (a self-report variable), 1 if health status were medium, 0 if otherwise			57 (19)
**Poor health**	Indicated the health status of each household head (a self-report variable), 1 if health status were poor, 0 if otherwise			12 (4)
**Age**	Indicated the age of each household’s head	51.32	14.39	
**Education**	Indicated the education years of each household’s head	7	4.9	
**past inpatient**	Indicated the family’s utilization of inpatient services in the past: 1 if services had been used, 0 if not			90 (30)
**Future inpatient**	Indicated the family’s utilization of inpatient services in the future : 1 if they would be utilized, 0 if not			33 (11)
**Drug users**	Indicated the number of family members using any medicine regularly			126 (42)
**Disable member**	Indicated the number of disabled people in each family			6 (2)
**under 5**	Indicated the number of children under 5 yr of age old in each family			51 (17)
**Over 65**	Indicated the number of elderly people (over 65 yr) in each family			69 (23)
**Marriage**	Indicated the marriage status of each household’s head: 1 if married, 0 if otherwise			273 91)
**Employment**	Indicated the employment status of each household’s head: 1 if employed, 0 if otherwise			189 (63)
**Unemployment**	Indicated the unemployment status of each household’s head: 1 if employed, 0 if otherwise			54 (18)
**Retired**	Indicated if each household’s head were retired, 1 if retired, 0 if not			57 (19)

All of 300 administrated questionnaires were suitably completed. The mean number of family members was 3.67. On average, the households’ heads were 51.32 yr old. On average, the education degree of the household heads was middle school. Ninety-one percent of household heads were male and 9% of them were female. Overall, 91% of households’ heads were married, 63% of them were employed, 18% were unemployed and 19% were retired.

**Fig. 1:**
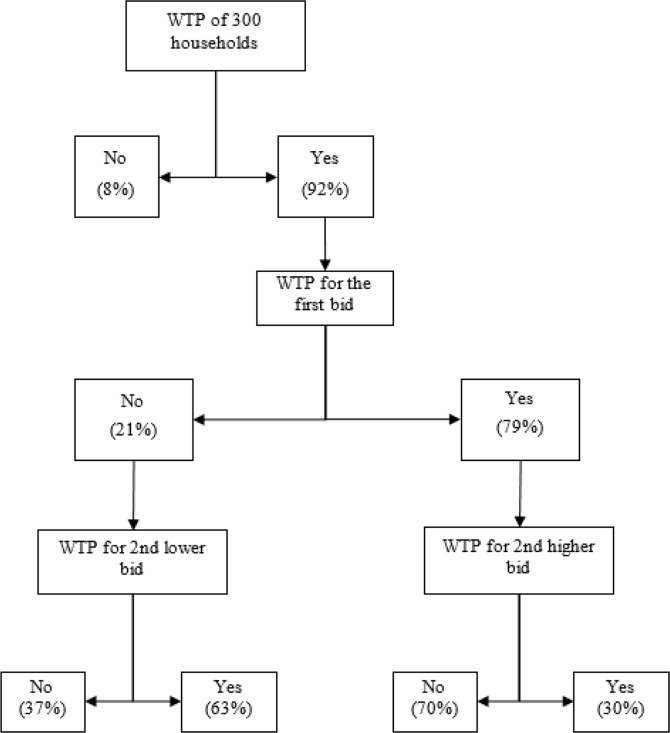
Statistical summary of the responses to double bounded dichotomous choice question

Out of 300 household heads, 290 were willing to join the health insurance scheme. Of 79% of them responded “yes” to the first bid and 21% responded “No”. Fig. 1 shows the summary of statistics of the responses to the double bonded dichotomous choice question

**Fig. 2:**
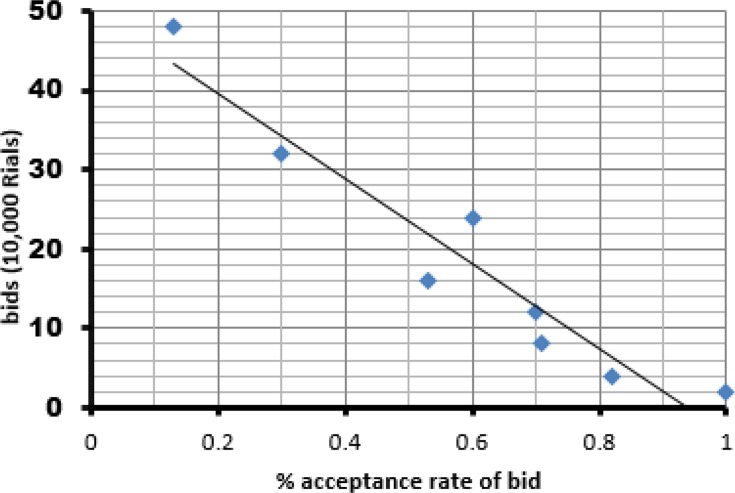
Household acceptance rate (%) and bids (10,000 Rial) (using double bounded dichotomous choice method)

Fig. 2 shows the relationship between the probabilities of accepting different bids. The downward sloping graph shows an inverse relationship between price and acceptance rate and indicates that the probability of accepting decreased by increasing the bids. The probability of accepting the bids ranged from 100% for the lowest bid to 13% for the highest bid.

The interval regression based on “yes” and “no” response of households in double-bounded method and OLS regression based on open-ended question method, performed using Stata ver. 11. The results of the regressions are presented in Table 2.

**Table 2:** The effect of explanatory variables on the willingness to pay of household heads

**Variables**	**Interval Regression**	**OLS**
**Gender**	1.78 (3.18)	−1.42 (2.26)
**Past – inpatient**	2.31 (1.68)	2.10 * (0.98)
**Future – inpatient**	3.29 (2.44)	1.38 (1.39)
**Under 5**	−3.98 * (1.99)	−1.98 (1.19)
**Over 65**	1.73 (2.23)	0.74 (1.31)
**Marriage**	−0.91 (3.25)	−0.79 (1.99)
**Income**	2.17* (1.09)	1.14 (0.65)
**Education**	0.67 (0.67)	0.50 (0.40)
**Disable members**	−0.28 (5.33)	3.45 (2.85)
**Drug- users**	1.52 (1.67)	0.39 (0.98)
**Age**	−0.06 (0.80)	−0.014 (0.047)
**Family size**	−3.05** (0.51)	−1.85 ** (0.30)
**Experience**	6.07* (3.17)	0.60 (1.69)
**Insured members**	−0.36 (0.83)	0.27 (0.48)
**Employed**	2.49 (2.08)	6.23* (2.65)
**Retirement**	1.07 (2.45)	0.23 (1.26)
**Good health**	−2.78 (1.68)	−1.33 (0.97)
**Middle health**	−3.74 (2.41)	−3.07 * (1.38)
**Poor health**	−8.42* (3.88)	−4.89 * (2.30)
**Constant**	28.17** (7.84)	17.58** (3.92)
**Sigma Constant**	10.00** (0.57)	

**, * significant at 1 and 5% respectively

Standard errors are in paranthesis

WTP of households on complementary health insurance depends not only on premium and benefits package but also on socio-economic and demographic characteristics of households.

The coefficients of income, utilization of inpatient services, experience of complementary health insurance and employed household heads were positive and significant; these indicated by increasing these variables WTP for complementary health insurance increased. The coefficients of family size, having less than 5 yr old child and middle and bad health status of household heads were negative meaning that by increasing these variables, WTP for complementary health insurance decreased.

The estimation of the WTP is demonstrated in Table 3. The findings showed the average of the WTP for the private health insurance per person per month was 199000 Rls (8 USD) CI (175000 – 222600 Rls) by DBDC and 115300 Rls (4.6 USD) by an open-ended question. Therefore, the prices were statistically significant.

**Table 3:** The mean of the willingness to pay of household heads per family member per month by DBDC and open-ended question methods

**Variable**	**Mean**	**Standard. Error**	**P-value**	**Confidence interval 95%**
**WTP by DBDC (10000 Rls)**	19.900	1.2	< 0.0001	17.500 – 22.26
**WTP by open-ended question method (10000 Rls)**	11.53	0.41	-	10.70 – 12.34

1 USD = 25000 Rls (At the time of study)

## Discussion

The mean of WTP for private complementary health insurance in Iran per person of family per month by DBDC and open-ended question method were respectively 199000 and 115300 Rls in 2014 (equivalent to 8 and 4.6 USD, respectively). We also examined the variables that might affect WTP of peoples. An important variable in decision of responded to pay is family size, by increasing family size the total sum of premium which household heads should pay increase. Thus, family size has a negative effect on WTP (10–15). Another significant variable is household income. The more the income, the less the ability of each household is to pay the premium. Therefore, income has a positive effect on WTP. This positive relationship between household income and their WTP accepted in earlier studies (10, 13, 16–19).

The relationship between health status and decision to purchase private health insurance coverage is an interesting finding. As discussed in the literature review section, people who reported they were in fair or poor health, had lower agreement to purchase health insurance coverage especially private health insurance in comparison to those reported excellent health (20, 21). Moreover, the result of our study indicated, that peoples with poor or medium health status have lower WTP in comparison to those having excellent health status.

The other significant variable is past utilization of inpatient services and past experience of having private health insurance meaning that people familiar with private health insurance were more WTP for health insurance.

Employment status was significantly linked with a higher WTP; directing employed household heads had more WTP for the health insurance. This finding is well established in the literature (13). In comparison with the unemployed or those with no regular income, employed people have regular incomes and health insurance is more affordable for them. Besides, Iranian insurers are more willing to sell health insurance coverage to groups of employers especially governmental companies. Maybe, this matter encourages employers to purchase health insurance coverage. Having at least one under 5 yr old child in family has negative effect on WTP.

The most significant and effective variables on the WTP of household were socio-economic and demographic variables affected by the macroeconomic and cultural status of the country. Then, again, these variables are difficult to manipulate by policy makers.

From policy point of view, the WTP value can be used as a premium in society. In Iran premium per person per month, for complementary health insurance is 140000 Rls (5.6 USD) until 170000 Rls (6.8 USD) and the mean amounts that estimated is 199000 Rls (8 USD) and lower bounded amount is 115300 Rls (4.6 USD), it is more than the real premium, this finding helps policy makers to increase the premium and increased their benefit package, and it may lead to more insured people. Number of insured people provides insurer a chance for risk pooling. More, according to the law of large number, the probability of insurer loss decreased. Therefore, potential increase of premium will blossom the insurance industry.

We tried to use appropriate modeling approaches and test their underlying assumptions. We selected a specific sub-sample of households, which had social coverage of health insurance, and they could voluntarily purchase complementary health insurance. In this study, we used a double bounded dichotomous choice format with open-ended question method to examine the WTP of households. The double bounded dichotomous choice method has significant statistical efficiency in comparison with other contingent valuation methods (3, 4). To avoid initial bias bid, which is common in the Contingent valuation method, we used four initial bids. The positive relationship between income and WTP for health insurance confirmed that the health insurance schemes were normal goods in Iran. Besides, this important finding proved the construct validity or internal validity of this study (2). Using open-ended question method is a suitable method for estimation low bounded of estimated amounts

This paper faced some limitation. First, finding household heads by telephone resulted in some difficulties. From the 600 households that we called, some of phone numbers were not valid or no one answered them after three times dialing, and 20 households did not participate in our survey. The sample of survey might not be a good representation of all households. Second, we considered the perceived quality of the services to be uniform for the entire study sample. Finally, the data received from the questionnaire survey provided only a snapshot of the households’ behaviors. Long-term prospective studies might provide better evidence for estimating the WTP of households and assessing the influence of different factors on the insurance purchase.

## Conclusion

This study set out to deliver the evidence on the WTP for complementary health insurance for households in Iran; the estimation amounts are more than the real premium in the country. It can offer the policy makers the opportunity to increase the premium and provide good benefits package for insured people of country then better risk pooling.

The variables affecting the household heads WTP are not directly affected by policy makers’ determinations. Overall analysis suggests that only national mandatory policies towards expansion of health insurance coverage may be enforced, however, it seems difficult.

## Ethical considerations

Ethical issues (Including plagiarism, informed consent, misconduct, data fabrication and/or falsification, double publication and/or submission, redundancy, etc.) have been completely observed by the authors.
